# 

**Published:** 1918-06

**Authors:** 


### ANNOUNCEMENTS

### The Forsyth Dental Infirmary for Children—Permanent Staff Appointments

A competitive examination of graduates in dentistry (of less than three years' standing) for appointments to positions on the Permanent Staff for full and one-half time service will be held early in June at the Infirmary.

Appointments will be made for one or two years as follows:

Full time service requiring operating five and one-half days a week at a salary of $1,000 a year.

One-half time service requiring operating six half-days a week, either forenoon or afternoon, at a salary of $400 a year.

These appointments will be made subject to satisfying the requirements of the Massachusetts State Board of Registration in Dentistry and to "qualifying" in the practical work of the clinics during one month's trial.

Members of this staff will be entitled to the advantages of reports and clinics by experts in the various branches of dentistry from different parts of the world in addition to the numerous regular clinics and lectures.

Operators after serving four months are eligible, by qualifying, for appointments in the special clinics where post graduate work is given.

The operators on this staff have the advantage of the clinics and lectures of the Post Graduate School of Orthodontia.

The Infirmary clinics provide unusual advantages in the various departments of the institution where Operative Dentistry, Orthodontia, Nose and Throat and Oral Surgery, Extracting, Novocaine Technic, Radiography, Pathological Diagnosis and Research Work, are continually carried on.

The average number of cases treated daily is more than 450 in all departments.

All material and necessary operating instruments will be furnished; up-to-date apparatus including electric engines, sterile instrument trays, fountain cuspidors, compressed air and the modern operating-room type of lavatories are available for use.

A diploma for service will be issued by the Trustees to each member of this staff who has completed this term of service in a satisfactory manner.

Applications for the above positions should be made not later than May 18th.

Information and the date of the examination will be furnished to those interested.—Harold DeW. Cross, D.M.D., Director, 140 The Fenway, Boston, Mass.

---

### General Clinic of the N. D. A.

Arrangements are sufficiently advanced to promise the members of the Association that the General Clinic will be one of the great features of the 1918 meeting.

In conference with officers of the National Dental Association, the committee in charge of the General Clinic carefully considered the nature of the clinic to be presented this year. After trying for the past few years new features in conducting the Clinic Program, it is the belief that a greater number will be served and benefited by holding a General Clinic, grouped into sections, namely: Operative, Prosthetic, Crown and Bridge Work, Orthodontia and Prophylaxis.

To make it national in character, the presidents of the different state societies, were requested to appoint two clinicians and two associates from his state society.

Up to date, thirty-nine state societies are represented and the remaining nine will be represented before the publishing of the Official Program.

Far away Alaska is sending two and two associates, and to make the Clinic more than national, in fact an allied affair, the Canadian Dental Association has promised ten of the best clinicians in the Dominion. In addition, there will be a few Unit Clinicians which will demonstrate principles that require more than two men.

It is safe to say that this clinic will be unique in the sense that every man on the program will either be present or be represented by his associate.—DON M. GALLIE, Chairman General Clinic.

---

### NATIONAL DENTAL ASSOCIATION

*Special Announcement of Hotels and Garages.* The National Dental Association will hold its Twenty-second Annual Meeting in Chicago, August 5-9, 1918. The headquarters will be at the Auditorium and Congress hotels, situated on Michigan avenue, corner of Congress street. All meetings, clinics and exhibits will be held in these two hotels, which are connected with an underground tunnel.

The important announcement at this time must be the warning, ''Reserve your rooms at once. Make reservations by mail direct to the hotel of your choice.

The following is a list of hotels and rates:

*Auditorium Hotel, Michigan Boulevard and Congress Street.* Single room without bath, $1.50 and $2.00 per day. Single room with bath, $2.50 to $4.00 per day. Double room without bath, $2.50 and $3.00 per day. Double room with bath, $4.00, $5.00 and $6.00 per day.

*Congress Hotel and Annex, Michigan Avenue and Congress Street.* Room, detached bath (one person), $2.00, $2.50, $3.00 per day. Room, private bath (one person), $3.00, $3.50, $4.00, $5.00, $6.00 per day. Room, detached bath (two persons), $3.00, $4.00, $5.00 per day. Room, private bath (two persons), $5.00, $6.00, $7.00 per day. Suites: Two connecting rooms, private bath (two persons), $6.00 to $10.00 per day. Three or four persons, $8.00 to $14.00 per day. Corner Suites: Parlor, bed room and private bath, $10.00 to $50.00 per day.—J. P. Buckley, Chairman Publicity Committee.

---

### National Association of Dental Examiners

The next meeting of the National Association of Dental Examiners will be held in Chicago, Ill., August 5 and 6 at the Auditorium Hotel. For further information address the Secretary, Dr. J. A. West, 417 Utica Building, Des Moines, Iowa.

---

### PREPAREDNESS LEAGUE OF AMERICAN DENTISTS—NEWS AND NOTES

*Communication from the President.* Who should receive the services of the members of the League? Every worthy man within the draft age whether he may or may not be in the service of his country at the time.

Let us forget all but the one thought—that we must do our utmost to help strengthen our great National Army. We must help make fighters to protect our flag and we must do it NOW. Tomorrow may be too late.

One of our members recently arose at 3 a. m. and worked until 5 that one of "our boys" might go away in comfort. It is noble work and thousands of instances similar to this one might be related. The pride we feel in the way our profession is meeting the crisis can not be expressed in mere words.

Every man you make dentally fit will fight for you in France.

HAVE AN ARMY OF YOUR OWN

*All League Service Must be Free.* It is well, perhaps, at this time to remind our members that all dental service rendered in the name of the League must be free. There can be no deviation from this rule without nullifying the great principle upon which our organization is founded.

We are rendering an inestimable service to our country and when we shall have completed our work, let it be said that we gave freely, gladly and with true patriotic spirit.

*Third Annual Meeting of the League.* Our third annual meeting will be held on August 7, in conjunction with the National Dental Association, Chicago, Ill.

We are arranging a program which will be of absorbing interest and urge every member who belongs to the N. D. A. to join us in the enjoyment of the good things we are preparing.—J. W. Beach.

*Communication from the Director General.* Regarding the editorial * which was enclosed with the letters you sent me to read, would say that it looks very much like German propaganda, although I presume it was printed in all innocence, but if the editor had stopped awhile to consider it, undoubtedly he would have adopted another form of criticism.

The Preparedness League is working under the supervision of the Surgeon-General's Office. That being the case, one must realize that before this Questionnaire was made up, very serious consideration was given the form of the questions, and each question was devised for a specific purpose. The general purpose of all questions is to help us in securing new members for the Preparedness League in order that the work may be increased to such an extent that the League may be of more efficient help to the Government.

*The Question of Charging for Service.* Regarding the matter of paying for dental service by those who are well able to pay. A dentist is asked to give but one hour a day to the registrants. He would, therefore, be giving but one hour of free dental service whether the man could pay or not, and this surely is not a great hardship on anyone. If the registrant pays for the service, then directly it ceases to be voluntary service on the part of

---

* Editorial from a western newspaper in which the "Questionnaire," asking dentists to join the League, was criticized.—Editor.

the dentist, and is therefore not Preparedness League work. There is no objection to a man who is able to pay for the work, going to his own dentist to have the work done and pay for it, but such service should not be recorded as Free Dental Service or as Preparedness League work, as it is work done strictly in a private capacity and is in no way connected with volunteer service. On the other hand, if any registrant, however well able to pay, is willing to accept free service at the hands of the Preparedness League, it seems very little for the League to take care of him in comparison with the man offering his life for our protection, and I should dislike very much to hear that any dentist would refuse volunteer service under these circumstances.

*Continuous Appointments not Practicable.* Making appointments with individual members of the drafted men, is, I think, not practicable for the reason that it is only through organized channels that the dentists offering their services, can be assigned their fair proportion of the work. If work was continued on each mouth until it was put in good condition, it would work out to the advantage of the few and the disadvantage of the many. If a registrant needs a second or third appointment to make his mouth dentally fit, refer him back to the League's Headquarters for such appointments, and if time permits, there is no doubt but that he will be taken care of; meanwhile, those who come after him are not being neglected.

On the other hand, if a registrant requires a second or third appointment and the dental operator is interested enough in his case to finish the work, then additional appointments may be made with the registrant providing they do not interfere with the time he has already pledged the League. In other words, only one hour is to be given each registrant unless other provision has been made for him either by the dental operator or by the Preparedness League. The *once over* for each registrant is the important thing to be borne in mind.

*Broken Appointments.* When appointments, either first or second, are not kept, the result, of course, is loss of time. When this happens it is unfortunate and must be considered a part of our personal sacrifice, as there seems to be no way to guard against it. New appointments should not be given those who break their first appointments, but they should be referred back to the League's Headquarters and sent back to the State Director.—Chas. F. Ash.

*Form 3-C Cards.* Each dentist should chart the operations performed, sign his name and apply the Stickers furnished by each State Director or County Director. If the operator has no Sticker, cross out the name of the selective on the address side and write Lieut. W. A. Heckard, D. R. C., 50 East 42d Street, New York City, across the card and mail at once.

*Form is Card.* When the work on each registrant is finished it should be recorded on Form 18 Card and when this Form 18 Card is filled up and duly signed it should be dropped in the box and sent back to the State Director. Each Card takes care of itself and records work done to date.—R. Ottolengui, Director of Publicity.

